# A Study of the Protective Properties of Iraqi Olive Leaves against Oxidation and Pathogenic Bacteria in Food Applications

**DOI:** 10.3390/antiox6020034

**Published:** 2017-05-17

**Authors:** Ammar B. Altemimi

**Affiliations:** Department of Food Science, College of Agriculture, University of Basrah, Basrah 61004, Iraq; ammar.ramddan@uobasrah.edu.iq or ammaragr@siu.edu

**Keywords:** Olive leaf, TBA, antioxidant, antimicrobial, coliform

## Abstract

There is an ancient and prodigious history of olive trees because of their nutritional, medicinal, and traditional uses. Intensive studies have been conducted on olive leaves because they have many positive and beneficial effects for human health. In this study, different solvents were used to examine the olive leaves for their antioxidant and antimicrobial activities and their possible food applications. The obtained results showed that the amounts of phenolic compounds of the olive leaf were 190.44 ± 0.50, 173 ± 1.72, 147.78 ± 0.69, and 147.50 ± 0.05 mg gallic acid/g extracts using methanol, ethanol, diethyl ether, and hexanol, respectively. The statistical analysis revealed that there was a significant difference in the phenolic contents in terms of the used solvents. The stability of the olive leaves extraction was also studied and the results indicated that increasing the storage temperature could negatively affect and encourage the degradation of the phenolic compounds. Furthermore, the olive leaf extraction was applied to raw sheep meat slides at 0.5%, 1.5%, and 2.5% (w/v) in order to test its antioxidant and antimicrobial effects. The results obviously showed that the sample treated with 2.5% olive leaf extract had the significantly (*p* < 0.05) lowest Thiobarbituric Acid (TBA) values of 1.92 ± 0.12 (mg Malonaldehyde MDA/kg) throughout 12 days of cold storage. Moreover, the results showed that the sample, which was treated with 2.5% olive leaf extract, had low total bacterial count and total coliform bacteria (6.23 ± 0.05, 5.2 ± 0.35 log colony forming unit (CFU)/g, respectively) among the control, 0.5%, and 1.5% olive leaf treated samples throughout 12 days of storage. The phenolic extracts from the olive leaf extract had significant antioxidant and antimicrobial activities, which could be used as a source of potential antioxidant and antimicrobial agents.

## 1. Introduction

Olive trees are known as trees of the subtropical region, which can survive and live for a few decades. The original homeland of olive trees is the Mediterranean region, including Iraq [1]. The estimated area of planted olive trees is approximately eight million hectares. In addition, many studies pointed out that the cultivation of olive trees began around 3500 years ago [2]. The olive leaf is one of the tree byproducts, which are obtained through either the pruning and harvesting process or fall due to different climatic factors. There is an ancient and prodigious history of olive trees because of their nutritional, medicinal, and traditional uses [3]. An essential part of the Mediterranean diet is olive products because of their ability to modulate and control the oxidative balance in vivo; indeed, they can monitor and inhibit pathogenic bacteria [4,5].

Lipid oxidation has been one of the major concerns of the scientific community for centuries. Scientists are persistently looking for antioxidants that will adequately work against oxidation in both fats and oils [6]. Synthetic antioxidants are used in most countries, and their usage level is regulated and the safety of the compounds has been tested based on long-term toxicity studies. Moreover, most of the synthetic antioxidants are stable either under processing conditions or under the storage status of oils and fats [7]. However, these synthetic antioxidants can cause carcinogenic and toxicological effects [8]. There are no decisive results on the safety of these materials, thus utilizing antioxidants from natural sources has arisen throughout the world [9].

There is an intensive investigation of olive leaves because of their many positive and beneficial effects for human health. For instance, olive leaves can work as anti-inflammatory, anti-microbial, anti-diabetic, anti-atherosclerotic, and anti-carcinogenic agents [10,11]. Nowadays, people are consuming and taking olive leaf extracts that can be in either liquid or capsule form. Oleuropein is considered one of the most abundant phenolic compounds in olive leaf extracts [12]. In fact, Oleuropein possess a spectacular effect against many foodborne pathogens such as *Staphylococcus aureus*, *Helicobacter pylori*, and *Campylobacter jejuni* [11]. Haddadin [13] found that olive tree leaves possessed large amounts of phenolic compounds such as vannilin, vannilic acid, verbascoside, luteolin-7-glucoside, and catechin; and their qualities were similar to those that are found in olives and other products derived from them, thus possessing the ability to prevent oxidation in food.

The main objectives of this study are; (1) to investigate the antioxidant and antimicrobial properties of olive leaf extract (OLE), (2) to determine the olive leaf extract chemical composition, and (3) to monitor the effects of using olive leaf extract on the quality of raw sheep meat slides.

## 2. Materials and Methods

### 2.1. Preparation of the Olive Leaf Extracts

Olive leaves were collected from an olive orchard located in Najaf province, Iraq. They were placed in polyethylene bags and kept in the refrigerator until use, and then the olive leaves were characterized by specialists at the Department of Horticulture, College of Agriculture, University of Basra. The olive leaves were cleaned and properly washed from extraneous matter. Then, they were dried in a hot air-oven for 48 h at 37 °C. A powder form of the dried olive leaves was obtained after crushing them in a blender. Thereafter, the olive leaf extracts were prepared according to Pereira et al. [10] by using the mixed solvents as follow:

Ethanol/H_2_O (80:20 v/v %)
Methanol/H_2_O (80:20 v/v %)
Diethyl ether/H_2_O (80:20 v/v %)
Hexanol/H_2_O (80:20 v/v %)

Five grams of powdered olive leaves was weighed and placed in a 500 mL beaker with the addition of 200 mL of each solvent separately. The solvent extraction process was performed at a temperature of 38 °C using a stirrer speed of 180 rpm for 20 min followed by filtration using Whatman no.1. Centrifugation was performed at 5000 r/min for 15 min, and then the filtrates were evaporated at 38 °C using a rotary evaporator for 4 h. The crude extracts were kept in dark containers in the refrigerator before use.

### 2.2. Determination of Total Phenolic Compounds

The total phenolic compounds were measured according to Lako et al. [14] with slight modifications. One mL of crude olive extract was mixed with 20 mL of (DMSO) dimeyhyl sulfuoxide. Afterwards, 2.5 mL of Folin-Ciocalteu was added with the addition of 10 mL of distilled water. The mixture was left for 2.5 min at room temperature. Then 2 mL of sodium carbonate (7.5%) was added, and the mixture was kept for one hour at room temperature in a dark place. The absorbance was read at a wavelength of 725 nm, and it was compared to a calibration curve prepared with known amounts of Gallic acid (Roth, Karlsruhe, Germany). The results were expressed as mg Gallic acid/g extracts.

### 2.3. Ferric Thiocyanate 

The antioxidant activity of the olive leaves was measured according to Elmastas et al. [15], which included measuring the effectiveness of the anti-oxidation of linoleic acid by mixing 1 mL of the crude extract with 4 mL of ethanol (95%), 4.1 mL of linoleic acid (2.5%) in ethanol, and 8 mL of phosphate buffer (0.05 M, pH 7). The mixture was incubated at a temperature of 45 °C for 24 h, and then 0.1 mL of this mixture was added to 9.7 mL of ethanol (75%), 0.1 mL of ammonium thiocyanate (30%), and 0.1 mL of ferrous chloride (0.02 M). All conditions were the same for preparing the control sample except for mixing 1 mL of distilled water instead of the sample extracts. The synthetic antioxidant butylated hydroxytoluene (BHT) was used as a model for comparison. The absorbance was measured at a wavelength of 500 nm using a UV-Spectrophotometer. The percentage of antioxidant activity was calculated as follows:

% Antioxidant activity = 100 − (A/B) × 100
A: Absorbance of thesample
B: Absorbance of thecontrol

### 2.4. Ferric Reducing Antioxidant Power

The antioxidant ability of the olive leaves was determined according to [16]. One mL of olive leaf extract was dissolved in 1 mL of distilled water, and 2.5 mL of K_3_Fe(CN)_6_ (1% w/v) with 2.5 mL of 0.2 M phosphate buffer (pH 6.6). The mixture was kept at 50 °C for 20 min, and then about 22.5 mL of trichloro acetic acid (10% w/v) was added. Afterwards, centrifugation at 3000 rpm for 10 min was performed in order to obtain an upper layer (2.5 mL). Then 2.5 mL of distilled water and 0.5 mL of FeCl_3_ (0.1%, w/v) were mixed with the obtained upper layer. All conditions were the same for preparing the control sample except for mixing 1 mL of distilled water instead of the sample extracts. The synthetic antioxidant (BHT) was used as a model for comparison. The absorbance was measured at 700 nm using a spectrophotometer. The ferric reducing antioxidant power was calculated as follows:

% Ferric reducing antioxidant power = 100 − (A_s_/A_c_) × 100
A_s_ = absorbance of the sample
A_c_ = absorbance of the control

### 2.5. Free Radical Scavenging Activity 

Firstly, the 1,1-diphenyl-2-picrylhydrazyl (DPPH) solution was prepared by dissolving 2 mg of this indicator in 100 ml of methanol. Secondly, the DPPH solution (3 mL) was added to 1 mL of olive leaf extract and was then mixed gently according to [17]. The mixture was incubated in a dark place for 30 min. The control sample was prepared by mixing 1 mL of methanol with 3 mL of the prepared DPPH solution. The synthetic antioxidant (BHT) was used as a model for comparison. The absorbance was measured at 517 nm for each sample using a spectrophotometer. The inhibition of DPPH activity was calculated using the following formula:

% Inhibition of DPPH activity= [A_c_ − A_s_/A_c_ ] × 100
A_c_: absorbance of the control
A_s_: absorbance of the sample

### 2.6. Studying the Stability of the Olive Leaf Extract

The preliminary results indicated that methanol extraction showed the highest values for the tested performance among the used solvents. For this reason, methanol extraction was used in the remainder of the experiments. The storage times and temperatures for the olive leaf were tested to ensure that there was minimal change for the total phenolic compounds, in order to avoid degradation of the phenolic compounds. Concentrated olive leaf extracts were stored at −18 °C, 5 °C, 25 °C, and 35 °C for 75 days. The total phenolic compounds were monitored every 15 days.

### 2.7. Determination of the Olive Leaf Extract Profile

The reversed phase HPLC with silica-based C18 was used as the stationary phase for determination of oleuropein, tyrosol, and verbascoside from the olive leaf extract. The mobile phase was a mixture of water and acetonitrile (80/20 volume ratio) containing 1% acetic acid at a flow rate of 1.0 mL/min. Oleuropein, tyrosol, and verbascoside in the crude olive leaf were identified by comparing their retention times with the corresponding standards [18].

### 2.8. Application of the Olive Leaf Extract on the Sheep Meat Slides 

#### 2.8.1. Preparation of Sheep Meat Samples

The sheep meat was obtained from local markets in Basra province. The sheep meat was cut into different sizes of slides (approximately 15 × 15 cm^3^). The weight of the sheep meat slides was about 10 g for each slide. The sheep meat slides were divided into five batches; three batches of sheep meat slides were immersed in solutions containing 0.5%, 1.5%, and 2.5% OLE (w/v) in a 1:1 ratio (sheep meat:distilled water w/v) for 15 h at 5 °C. After treatment, the samples were drained. The fourth batch was used as a control sample by immersion in distilled water. Ziploc bags were used to keep the sheep meat slides in five portions and were stored at 5 °C for 12 days.

#### 2.8.2. pH Determination of the Sheep Meat Slides 

The pH of the sheep meat slides was estimated according to Aytul et al. [19]. Approximately 2–2.5 g of sheep meat slides were weighed and mixed with distilled water (1:10 w/v). Centrifugation was performed at 12,000 rpm for 4 min in order to homogenize the mixture of sheep meat slides and distilled water. The pH of treated and non-treated samples was measured in triplicate by a pH meter.

### 2.9. Determination of Oxidative Stability of the Sheep Meat Slides 

#### 2.9.1. Thiobarbituric Acid (TBA) Assay

The TBA assay was carried out according to Bekhit et al. [20] with slight modifications. Firstly, the solution of TBA (0.45 %) and trichloroacetic acid (TCA) (prepared in 0.25 N HCl) was prepared. Then 2.5 grams of treated sheep meat slides (0.5%, 1.5%, and 2.5% w/v) were mixed and homogenized at 10,000 rpm for 5 min in a beaker containing 25 mL of the prepared solution of TBA and TCA. Then, in order to develop a pink color, approximately 5 mL of the homogenized sample was placed in a water bath at 100 °C for 12 min. The boiled samples were cooled under tap water. Thereafter, centrifugation at 5000 rpm for 15 min was performed. The whole procedure was repeated by using distilled water instead of the olive leaf extract (control sample), while BHT was used for comparison. Finally, the absorbance of the treated samples was measured at 532 nm using a spectrophotometer. The TBA value was calculated as follows: TBA [mg Malonaldehyde (MDA) equivalent/kg sheep meat] = A × 7.8, where A: the absorbance.

#### 2.9.2. Antimicrobial Activity of the Olive Leaf Extract on Sheep Meat Slides 

The antimicrobial activity of the olive leaf extract was determined according to Andrews [21] with slight modifications. Approximately 10 g of treated sheep meat slides (0.5%, 1.5%, and 2.5% w/v) was mixed and homogenized with 90 mL of 0.1% sterile peptone water under sterilized conditions for 2 min at 25 °C. The serial dilution of the mixture was made using 0.1% peptone water. The diluted samples were transferred to a petri dish containing nutrient agar and MacConkey agar in order to determine both the total count bacteria and total coliform bacteria, respectively. Microbial counts were expressed as log_10_ CFU/g of the sample. 

### 2.10. Statistical Data Analysis

The SPSS statistical software program (SPSS for Windows version 17, Spss Inc., Chicago, IL, USA) was used to analyze the data. Data were expressed as mean ± standard deviation (SD). The obtained results were considered significant at *p* < 0.05.

## 3. Results and Discussion 

### 3.1. Total Phenolic Contents 

**Figure 1:**
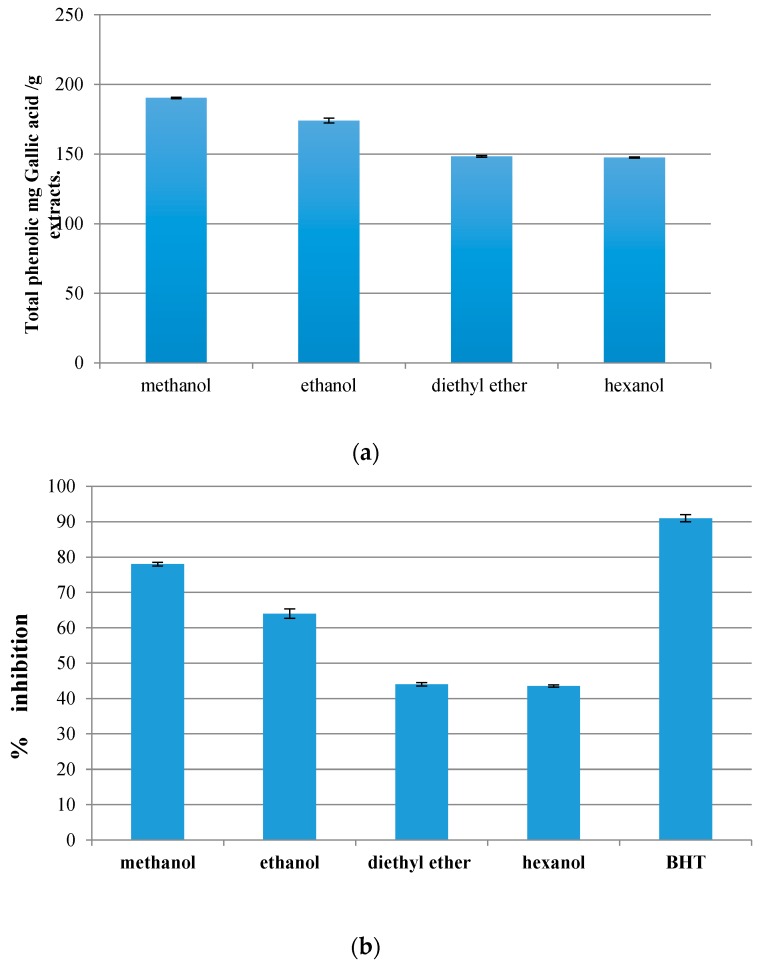

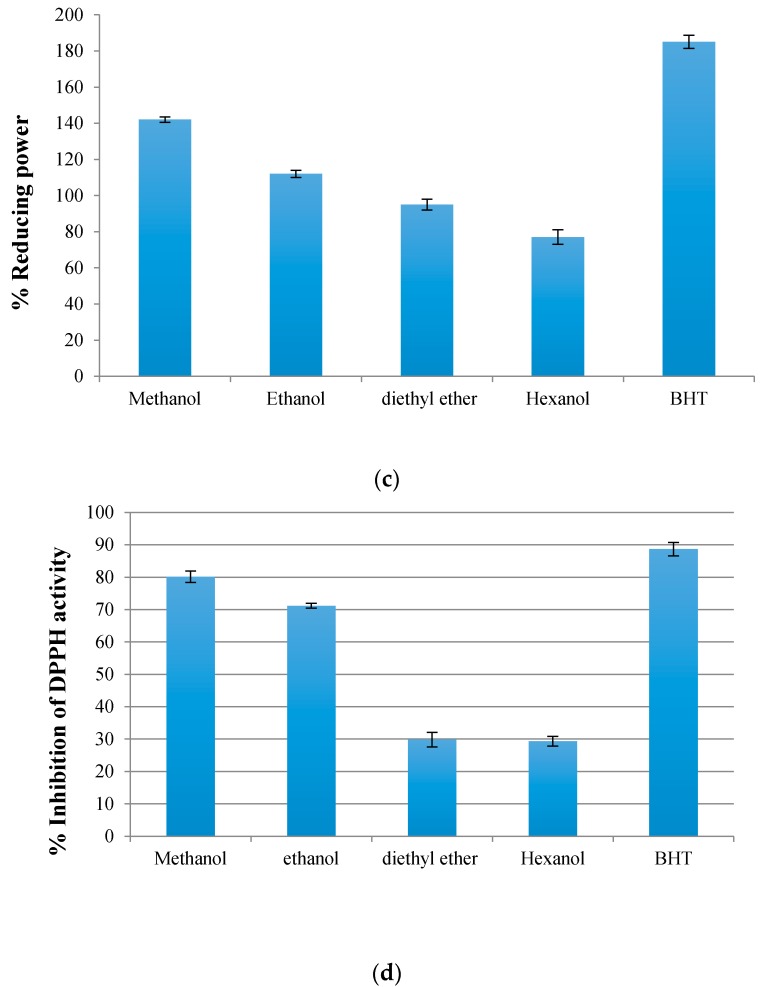
(**a**) Total phenolic content of the olive leaf. (**b**) Percentage of inhibition of linoleic acid peroxidation. (**c**) Percentage of reducing power of the olive leaf extracts. (**d**) DPPH radical scavenging activity of different plant extracts.

The total phenolic content of the olive leaves was expressed and presented in terms of mg gallic acid/g extracts (Figure 1a). The obtained results showed that the amount of phenolic compounds of the olive leaf were 190.44 ± 0.50, 173 ± 1.72, 147.78 ± 0.69, and 147.50 ± 0.05 mg gallic acid/g extracts using methanol, ethanol, diethyl ether, and hexanol, respectively. The statistical analysis revealed that there was a significant difference in the phenolic contents in terms of the solvents used. Among all the solvents, methanol extraction gave the highest amount of phenolic compounds followed by ethanol extraction, while the lowest amount of phenolic compounds was obtained using either diethyl ether or hexanol extraction. These results were in agreement with Zhou and Yu [22], who mentioned that using polar solvents could surely enhance and provide the highest amount of phenolic compounds in the examined plants.

### 3.2. Antioxidant Properties

The antioxidant property of the olive leaf was estimated using diverse methods in order to obtain accurate results.

#### 3.2.1. Ferric Thiocyanate Method

The results indicated that methanol extraction of the olive leaves showed the strongest extraction (78% ± 0.5), while the percentage of inhibition of the ethanol extraction was (63% ± 1.32) (Figure 1b). Both diethyl ether and hexanol gave the lowest percentage of inhibition (44% ± 0.5 and 43.92% ± 0.34, respectively). These results confirm the superior ability of methanol for the extraction of phenolic compounds, which own the effectiveness of the anti-oxidant and thus give it a significantly higher effectiveness than other solvents. Furthermore, the statistical analyses found that the percentage of inhibition of the methanol extract and ethanolic extract were significantly (*p* < 0.05) lower than the percentage of inhibition of BHT (92.17% ± 1.04). These results were consistent with that indicated by Ilango et al. [23] in their study of the antioxidant activity of the leaf plant *Adhatoda zeylanica* using the methanol extract.

#### 3.2.2. Total Reducing Power 

The reducing power capability of the olive leaf extract compared with BHT is examined in Figure 1c. The reducing power of the methanol extraction of the olive leaf was found to be remarkable and significant (*p* < 0.05) compared to the other solvents’ extraction. The percentage of reducing power was 143.3% ± 1.52, 114.13% ± 1.80, and 183.8% ± 1.08 for the methanol extraction, ethanolic extraction, and BHT, respectively. Furthermore, the statistical analyses found that there was no significant difference between diethyl ether extraction and hexanol extraction. This result was in agreement with Aliyu et al. [24] who measured the reductive power of the methanol extract of the *Bauhinia rufescens* leaf, and found that there was a potential effect for this extract to be used as a reducing factor by emitting a hydrogen atom and thus quenching free radicals. Moreover, the reductive capability depended on the ability of the plant extract and BHT to convert Fe^+3^ to Fe^+2^, thus giving the highest values of the reducing power. 

#### 3.2.3. DPPH Radical Scavenging Assay 

First, the antioxidant activity of the olive leaf extracts was investigated using the DPPH scavenging method by measuring the total antioxidant capacity. In this study, the effectiveness of the olive leaf extracts using different solvents was compared with the synthetic antioxidant BHT. The methanol extract of the olive leaves had a significant effect (80.166% ± 1.75) on the inhibition of DPPH activity among all the solvents used (Figure 1d). In contrast, BHT exhibited the highest antioxidant activity (88.67% ± 2.08) by decolorizing the DPPH reagent and then changing the absorbance reading positively. Both diethyl ether and hexanol extracts were found to be less active against DPPH (29.83% ± 2.25, 29.33% ± 1.53), respectively. This finding was similar to that of Gkanatsiou et al. [8], who found that more polar solvents had the ability to penetrate and dissolve more polar phenolic compounds from the olive leaf. The obtained results were not in an agreement with Hamad [25], who reported that the percentage of inhibition of the Saudi olive leaf was (51.72% ± 0.66) using ethanol extraction. The reason may be ascribed to the differences between the cultivars used.

### 3.3. Comparison between Total Phenolics Compounds’ and Antioxidant Activities’ Methods Performance

The preliminary results indicated that methanol extraction showed the highest values for the tested performance among the solvents used. For this reason, the correlation between total phenolic compounds’ and antioxidant activities’ methods performance depended on the methanol extraction. The researchers measured the value of R^2^ in order to determine if the data exhibited a linear relationship. A low correlation (R^2^ = 0.157) between the phenolic compounds and the percent inhibition of DPPH activity was indicated; this low correlation was also mentioned by Rezaeizadeh et al. [26], who explained and suggested that the decreasing radical scavenging values may be ascribed to the weakness of the DPPH oxidizing agent when the highest amount of phenolic compounds are present.

A weak correlation (R^2^ = 0.355) was shown between the values of phenolic compounds of the olive leaf extract and the ferric thiocyanate assay. Thus, the value of R^2^ of the ferric thiocyanate assay and the total phenolic content was found to have an unacceptable fit line. This obtained result may be related to the purification process and concentration of phenolic compounds during the preparation of the olive leaf extract.

The correlation between the total phenolic content and the reducing power activity was assigned. The regression analysis (*y* = 1.6429*x* − 143.14) showed a strong correlation (R^2^ = 0.994) between the total phenolic content and the reducing power activity. This result confirms that the plant extracts with the highest amount of phenolic content will be more effective at scavenging free radicals. These findings were in an agreement with Altemimi et al. [27] and Fernandez-Gutierrez [28], who discovered that the phenolic profile was considered very important to show the relation between the abovestated categories.

### 3.4. Stability of the Olive Leaf Extract

**Figure 2:**
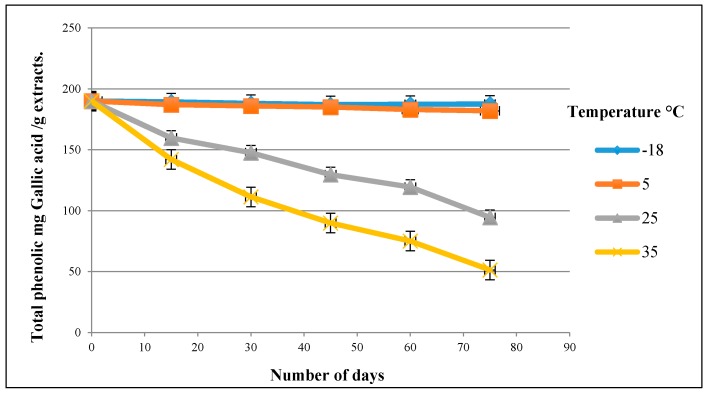
Stability of the olive leaf extract at different temperatures over a period of 75 days.

Figure 2 shows the estimated variation of the total phenolic content of the methanol extraction of the olive leaves. The differences between the stored samples were investigated using the *T*-test at 95% confidence level. The statistical analysis found that there was no significant difference (*p* > 0.05) between the stored samples at the freezing temperature at −18 °C nor the cooling temperature at 4 °C for up to 75 days. However, the obtained results revealed that there was a significant difference (*p* < 0.05) between both 25 °C and 35 °C. The total recovery of phenolic compounds for both 25 °C and 35 °C was 49.75% and 27%, respectively, when the olive leaf extract was stored for 75 days. This result indicated that increasing the storage temperature could negatively affect and encourage the degradation of the phenolic compounds. These findings were in an agreement with Obied et al. [29] who studied the effect of the storage condition on the phenolic compound and antioxidant activity of olive mill waste. 

### 3.5. Olive Leaf Extract Profile

**Figure 3:**
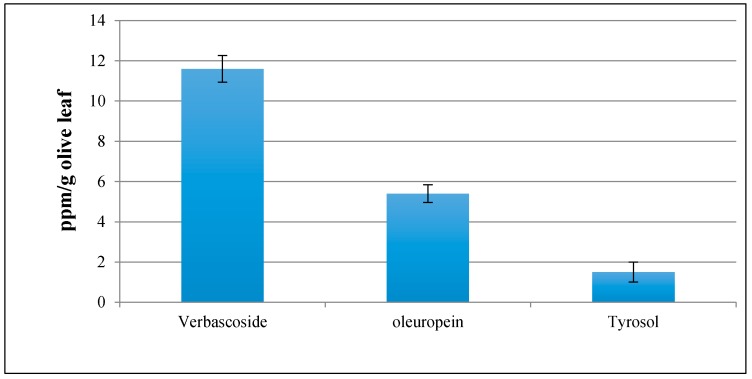
Olive leaf extract profile.

**Figure 4:**
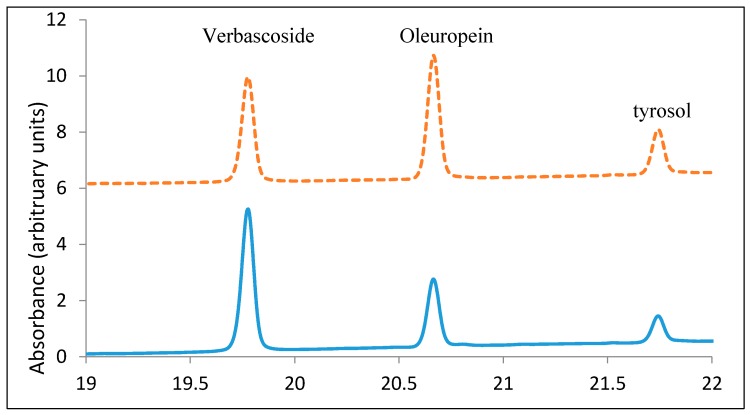
HPLC chromatogram of the olive leaf methanol extracts and the standards.

The methanol extracts of the olive leaves were examined by HPLC with ion trap mass spectrometry (IT-MS) and three major compounds (verbascoside, oleuropein, and tyrosol) were identified (Figure 3). The corresponding references of verbascoside, oleuropein, and tyrosol were used to compare the retention times with unknown phenolic constituents of the crude olive leaf extract, and then quantified all of them by using the calibration curves (Figure 4).

### 3.6. Application of the Olive Leaf Extract on Sheep Meat Slides

#### pH Determination of the Sheep Meat Slides 

First, the pH of the raw meat was estimated to be 6.44 (Table 1). The statistical analysis revealed that there was no significant difference (*p* > 0.05) between the control samples and all the treated sheep meat slides with either 0.5% or 1.5% olive leaf extracts through six days of cold storage. However, the sample treated with 1.5% olive leaf extract had a low pH value at eight days of cold storage compared to the control and 0.5% olive leaf extract treatment. In contrast, the sample treated with 2.5% olive leaf extract had significantly (*p* < 0.05) lower pH values (6.29 ± 0.05) during the whole 12 days of cold storage. 

### 3.7. Determination of Oxidative Stability of Sheep Meat Slides 

#### Thiobarbituric Acid (TBA) Assay

The lipid oxidation was determined using TBA analysis as an index (Table 2). The statistical analysis showed that there was no significant difference (*p* > 0.05) between the control samples and all the treated sheep meat slides with either 0.5% or 1.5% olive leaf extract through four days of cold storage. However, the sample treated with 1.5% olive leaf extract had a low TBA value at eight days of cold storage compared to the control and 0.5% olive leaf extract treatment. Increasing TBA values using either 0.5% or 1.5% olive leaf extract could be related to an extremely lower concentration of antioxidant material within each sample. In contrast, the sample treated with 2.5% olive leaf extract had significantly (*p* < 0.05) lower TBA values (1.92 ± 0.12 mg MDA /kg) throughout the 12 days of cold storage. Moreover, the results revealed that the BHT treatment exhibited the lowest TBA values (1.44 ± 0.17) mg MDA /kg among the olive leaf concentrations except for 2.5%. This result was similar to that discovered by Verma and Sahoo [30]. Scientists said that if the TBA value was less than 2 mg MDA/kg meat, the plant extracts would be accepted as good quality.

### 3.8. Antimicrobial Activity of the Olive Leaf Extract on Total Count Bacteria of the Sheep Meat Slides 

Table 3 shows the effect of different concentrations of the olive leaf extract on the total count bacteria of the refrigerated stored sheep meat slides. The preliminary investigation measured the total count bacteria of the fresh sheep meat slides, which was 3.5 log CFU/g. After that, the total count bacteria dramatically increased during 12 days of storage at 5 °C in both the control sample and the sheep meat slides treated with olive leaf extracts. The statistical data analysis showed that there was no significant difference (*p* > 0.05) between either the control sample and the 0.5%, 1.5%, and 2.5% olive leaf extracts during three days of cold storage, while the 1.5% and 2.5% olive leaf extract were statistically significant compared to control sample and the 0.5% olive leaf extract during the same period of time. There was significant differences between the 1.5% and 2.5% olive leaf extracts at 6, 9, and 12 days of the cold storage condition. Moreover, the results showed that the sample treated with the 2.5% olive leaf extract had low microbial counts (6.23 ± 0.05 log CFU/g) compared to the control, 0.5%, and 1.5% olive leaf extract treated samples after 12 days of the cold storage condition. This finding was in an agreement with that of Ahmed et al. [31], who found that using increasing concentrations of olive leaf extracts as antibacterial compounds had a powerful effect in controlling the microbial load of raw peeled shrimp.

### 3.9. Antimicrobial Activity of the Olive Leaf Extract on Total Coliform Bacteria of the Sheep Meat Slides 

The initial determination of total count bacteria of the fresh sheep meat slides was 1.5 log CFU/g, as shown in Table 4. Thereafter, the results showed that the total coliform bacteria spectacularly increased during 12 days of storage at 5 °C in both the control sample and the sheep meat slides treated with olive leaf extracts. The statistical analysis showed that there was no significant difference (*p* > 0.05) between the control sample and the 0.5% olive leaf extract during 12 days of cold storage, whereas both the 1.5% and 2.5% olive leaf extract were statistically significant compared to the control sample and the 0.5% olive leaf extract during the same period of time. Furthermore, there was no significant difference (*p* > 0.05) between the 1.5% and 2.5% olive leaf extract during 3 and 6 days of the cold storage condition. However, the results found that the total coliform bacteria was statistically significant (*p* < 0.05) between the 1.5% and 2.5% olive leaf extracts at 9 and 12 days of the cold storage condition. The results showed that the sample treated with 2.5% olive leaf extract had low microbial counts (5.2 ± 0.35 log CFU/g) compared to the control, 0.5%, and 1.5% olive leaf extract treated samples during the entire storage period of 12 days. Similar results were observed and reported by Rahman et al. [32].

## 4. Conclusions 

Methanol extraction produced the highest amount of phenolic compounds and antioxidant activity followed by ethanol extraction, diethyl ether extraction, and hexanol extraction. The phenolic extracts from the olive leaves possessed and exhibited reliable antioxidant and antimicrobial effects, which could be used as a source of potential antioxidant and antimicrobial agents. This study exhibited useful and beneficial information; therefore this obtained knowledge will be very helpful for the further exploitation and application of this resource.

## Figures and Tables

**Table 1:** Effect of the olive leaf extract on pH values of the sheep meat slide.

Treatment	Number of Days *			
	2	4	8	12
Control	6.53 ± 0.02 ^a^	6.55 ± 0.02 ^a^	6.66 ± 0.03 ^a^	6.75 ± 0.03 ^a^
0.5 % olive leaf	6.52 ± 0.03 ^a^	6.54 ± 0.02 ^a^	6.62 ± 0.03 ^a^	6.74 ± 0.02 ^a^
1.5 % olive leaf	6.52 ± 0.02 ^a^	6.52 ± 0.02 ^c^	6.49 ± 0.03 ^b^	6.73 ± 0.04 ^a^
2.5 % olive leaf	6.39 ± 0.03 ^b^	6.36 ± 0.02 ^b^	6.32 ± 0.02 ^c^	6.29 ± 0.03 ^b^

* Means within each column with the same superscript letter (^a,b,c^) are not significantly different.

**Table 2:** Effect of the olive leaf extract on TBA values (mg MDA /kg) of the sheep meat slides stored at 5 °C for 12 days.

Treatment	Number of Days *			
	2	4	8	12
Control	2.24 ± 0.15 ^a^	3.76 ± 0.23 ^a^	4.68 ± 0.18 ^a^	5.75 ± 0.10 ^a^
0.5% olive leaf	2.27 ± 0.06 ^a^	3.73 ± 0.20 ^a^	4.69 ± 0.06 ^a^	5.76 ± 0.02 ^a^
1.5% olive leaf	2.23 ± 0.06 ^a^	3.72 ± 0.03 ^a^	4.09 ± 0.11 ^b^	4.92 ± 0.04 ^b^
2.5% olive leaf	1.27± 0.15 ^b^	1.33 ± 0.06 ^b^	1.69 ± 0.12 ^c^	1.92 ± 0.12 ^c^
BHT 0.02%	0.99 ± 0.12 ^c^	1.12 ± 0.16 ^c^	1.22 ± 0.21 ^d^	1.44 ± 0.17 ^d^

* Means within each column with the same superscript letter (^a,b,c,d^) are not significantly different.

**Table 3:** Effect of the olive leaf extract on total count bacteria (log CFU/g) of the sheep meat slides stored at 5 °C for 12 days.

Treatment	Number of Days *			
	3	6	9	12
Control	5.37 ± 0.15 ^a^	6.37 ± 0.21 ^a^	8.95 ± 0.13 ^a^	10.06 ± 0.16 ^a^
0.5% olive leaf	5.33 ± 0.36 ^a^	6.33 ± 0.25 ^a^	8.99 ± 0.31 ^a^	10.02 ± 0.27 ^a^
1.5% olive leaf	4.2 ± 0.1 ^b^	5.37 ± 0.15 ^b^	7.5 ± 0.17 ^b^	8.84 ± 0.03 ^b^
2.5% olive leaf	4.13 ± 0.12 ^b^	3.67 ± 0.15 ^c^	5.47 ± 0.25 ^c^	6.23 ± 0.05 ^c^

* Means within each column with the same superscript letter (^a,b,c^) are not significantly different.

**Table 4:** Effect of the olive leaf extract on total coliform bacteria (log CFU/g) of the sheep meat slides stored at 5 °C for 12 days.

Treatment	Number of Days *			
	3	6	9	12
Control	3.13 ± 0.32 ^a^	5.09 ± 0.21 ^a^	7.89 ± 0.4 ^a^	9.11 ± 0.23 ^a^
0.5% olive leaf	3.12 ± 0.36 ^a^	5.11 ± 0.41 ^a^	7.87 ± 0.31 ^a^	9.1 ± 0.37 ^a^
1.5% olive leaf	2.32 ± 0.21 ^b^	3.09 ± 0.13 ^b^	5.1 ± 0.31 ^b^	6.87 ± 0.27 ^b^
2.5% olive leaf	2.3 ± 0.31 ^b^	3.07 ± 0.15 ^b^	4.05 ± 0.29 ^c^	5.2 ± 0.35 ^c^

* Means within each column with the same superscript letter (^a,b,c^) are not significantly different.
